# The additive effect of the estimated glucose disposal rate and a body shape index on cardiovascular disease: A cross-sectional study

**DOI:** 10.1371/journal.pone.0331005

**Published:** 2025-08-21

**Authors:** Qinghua Wen, Xiaoyue Wang, Simin Li, Huanhuan Zhu, Fengyin Zhang, Chao Xue, Juan Li

**Affiliations:** 1 School of Nursing, Guizhou University of Traditional Chinese Medicine, Guiyang, China; 2 Public Health School, Zunyi Medical University, Zunyi, China; 3 Nursing School, Zunyi Medical University, Zunyi, China; 4 Department of Nursing, Guizhou Provincial People’s Hospital, Guiyang, China; Ferdowsi University of Mashhad, IRAN, ISLAMIC REPUBLIC OF

## Abstract

**Background:**

The glucose disposal rate (eGDR) and a body shape index (ABSI) are predictors strongly associated with cardiovascular disease (CVD) and outcomes. However, whether they have additive effects on CVD risk is unknown. This study aimed to investigate whether combined assessment of eGDR and ABSI could improve prediction of CVD risk.

**Methods:**

The current study used data from NHANES from 1999 to 2018 and included 14,237 participants. Receiver operating characteristic (ROC) curve was used to evaluate the performance of each indicator in predicting CVD. Machine-learning algorithms were applied to screen variables to adjust the model. Finally, the ROC curve, net reclassification improvement (NRI), integrated discrimination improvement (IDI), calibration curve and decision curve analysis (DCA) were used to evaluate the predictive performance of the combination of eGDR and ABSI.

**Results:**

The ROC curve showed that eGDR (C-statistics: 0.7255) and ABSI (0.7093) had the highest predictive performance. Among 14,237 participants, multivariate logistic regression showed that lower eGDR (≤6.448) and higher ABSI (≥0.086) significantly increased CVD risk (OR = 11.792, P < 0.05). The model adjusted by machine learning significantly improved CVD risk prediction (Model 3 vs. Model 1, C-statistics: 0.849 vs. 0.753). These findings were also consistent in the NRI (model 3 vs. model 1: 0.108), IDI (0.107), calibration curve, and DCA analyses. Subgroup analyses confirmed the robustness of these findings, with enhanced predictive performance particularly in younger populations.

**Conclusion:**

The eGDR and ABSI have potential additive effects on predicting CVD risk, and have excellent predictive performance, which can evaluate cardiovascular risk more comprehensively.

## Introduction

Cardiovascular disease (CVD) remains a leading cause of morbidity and mortality worldwide, placing a significant burden on public health systems [1]. Over the past three decades, the incidence and prevalence of CVD have continued to rise, particularly among the younger and in regions with increasing metabolic disorders [2,3]. According to global epidemiological data, approximately 523 million people suffer from CVD, leading to more 10 million deaths annually, with ischemic heart disease and stroke being the predominant subtypes, accounting for more than 66% of CVD-related fatalities [4,5]. Therefore, timely assessment of CVD risk is crucial in reducing the burden of cardiovascular disease and minimizing adverse cardiovascular events.

Numerous risk factors contribute to the development and progression of CVD, including traditional factors such as hypertension, dyslipidemia, diabetes, smoking, and obesity, as well as emerging metabolic and inflammatory markers [6,7]. To improve CVD risk prediction, various biomarkers and surrogate indices have been proposed. Among them, anthropometric and metabolic indices have been extensively studied for their predictive value in CVD outcomes [8,9]. These indices include a body shape index (ABSI), visceral adiposity index (VAI), atherogenic index of plasma (AIP), and lipid accumulation product (LAP) [10,11]. Additionally, insulin resistance-related markers, which have been confirmed to be strongly associated with CVD, include the triglyceride-glucose (TyG) index, homeostatic model assessment of insulin resistance, metabolic score for insulin resistance, and estimated glucose disposal rate (eGDR) [12,13]. While some studies have demonstrated the superior performance of these indices in predicting cardiovascular events, others have yielded inconsistent findings, and limited research has explored whether these indices have an additive effect on prognosis [12,14–16]. Furthermore, these indices primarily focus on specific or singular physiological pathways, which restricts their ability to provide a comprehensive evaluation of CVD risk [17,18]. Relying solely on a single predictor may fail to capture the multifactorial nature of CVD pathogenesis.

Given the limitations of individual predictive markers, integrating multiple indices may provide a more robust approach to assessing CVD risk [19]. By leveraging mature statistical methods, machine learning models, and visualization techniques, a more comprehensive evaluation of predictive efficacy can be achieved. Machine learning algorithms, including support vector machine-recursive feature elimination (SVM-RFE), extreme gradient boosting (XGBoost), and the boruta algorithm, have been successfully applied to feature selection and model construction [20,21]. These techniques enable the identification of the most relevant predictors and enhance the interpretability of multivariable models. In this study, we utilized a large, nationally representative cohort to systematically compare the predictive performance of various metabolic and anthropometric indices for CVD. Our objective was to determine whether a combination of these indices could outperform single markers in predicting CVD risk, ultimately improving risk stratification and guiding clinical decision-making.

## Methods

### Study data sources, ethics, population and design

**Fig 1:**
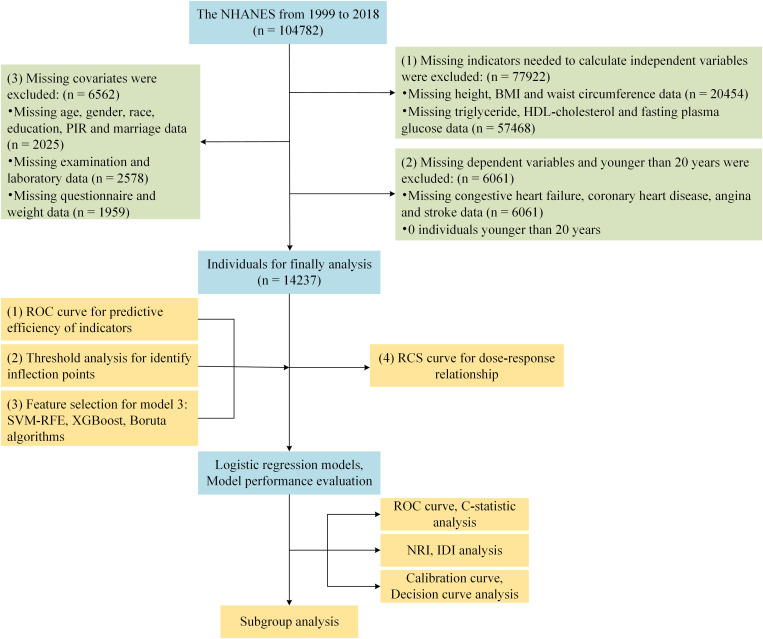
Flowchart of the study.

This study is a cross-sectional analysis based on publicly available data from the National Health and Nutrition Examination Survey (NHANES) (https://www.cdc.gov/nchs/nhanes/). All measures collected by trained personnel via standardized methods. This study was approval by the NCHS Research Ethics Review Board (ERB) (Protocol #2011–17). All participants consented to participate. Each participant provided written permission, which is available on the NHANES website. No minors were included in this study. In this study, we analyzed NHANES data collected between 1999 and 2018. The exclusion criteria included one of the following: (1) Without height, BMI, waist circumference, total triglyceride, HDL-cholesterol, fasting plasma glucose data (n = 77,922); (2) Without cardiovascular disease data (n = 6,061); (3) Without other covariates and weight data (n = 6,562). A final total of 14,237 patients were included (Fig 1).

### Assessment of cardiovascular disease-related indicators

In this study, we used commonly used cardiovascular disease-related indicators with good predictive performance in previous studies, including ABSI, body roundness index (BRI), cardiometabolic index (CMI), VAI, waist triglyceride index (WTI), LAP, AIP, TyG and eGDR [11,12,22]. These indicators are calculated using the following formula.

(1) The ABSI was based on waist circumference (WC) adjusted for height and weight [23]:


$$ABSI=\frac{WC(m)}{BMI{\left(kg/m^{2}\right)}^{2/3}\times height{\left(m\right)}^{1/2}}\mathrm{\backslash ]}$$

(2) The BRI was calculated using the following formula [24]:


$$BRI=364.2-365.5\times \sqrt{1-(\frac{{\left(WC(cm)/(2\pi )\right)}^{2}}{{\left(0.5\times height(cm)\right)}^{2}})}\mathrm{\backslash ]}$$

(3) The CMI was calculated using the formula [25]:


$$CMI=\frac{TG(mmol/L)}{HDL-C(mmol/L)}\times \frac{WC(cm)}{height(cm)}\mathrm{\backslash ]}$$

(4) The VAI was determined by the formula [26]:


$$\begin{array}{c} Males:VAI=\frac{WC(cm)}{39.68+(1.88\times BMI(kg/m^{2}))}\times (\frac{TG(mmol/L)}{1.03})\times (\frac{1.31}{HDL--C(mmol/L)})\\ {}[3pt]Females:VAI=\frac{WC(cm)}{36.58+(1.89\times BMI(kg/m^{2}))}\times (\frac{TG(mmol/L)}{0.81})\times (\frac{1.52}{HDL--C(mmol/L)}) \end{array}$$

(5) The WTI was calculated using the formula [22]:


WTI=WC(cm)×TG(mmol/L)\]

(6) The LAP was calculated as follows [27]:


$$\begin{array}{c} Males:LAP=(WC(cm)-65)\times TG(mmol/L)\\ Female:LAP=(WC(cm)-58)\times TG(mmol/L) \end{array}$$

(7) The AIP was calculated using the formula [28]:


$$AIP=log(\frac{TG(mg/dL)}{HDL-C(mg/dL)})\mathrm{\backslash ]}$$

(8) The formula for calculating the TyG is as follows [29]:


$$TyG=ln(\frac{TG(mg/dL)\times FPG(mg/dL)}{2})\mathrm{\backslash ]}$$

(9) The eGDR was calculated using the formula [30]:


$$\begin{array}{c} Hypertension:eGDR=21.158-(0.09\times WC(cm))-3.407-(0.551\times HbA1c(\%))\\ Non\text{-}hypertension:eGDR=21.158-(0.09\times WC(cm))-(0.551\times HbA1c(\%)) \end{array}$$

### Ascertainment of outcomes

All responses were collected by NHANES interviewers during structured interviews; no additional data were collected by the authors. The diagnosis of CVD was established by self-reported physician diagnoses obtained during an individual interview using a standardized medical condition questionnaire. The participants were asked, “Has a doctor or other health expert ever informed you that you have congestive heart failure/coronary heart disease/angina pectoris or heart attack (myocardial infarction)/stroke?” Congestive heart failure, coronary heart disease, angina pectoris, and stroke are also defined according to the problems of the corresponding diseases mentioned above. A person was regarded as having CVD if he or she replied “yes” to any of the above questions. NHANES mitigates self-report limitations via standardized interviews and medical record cross-validation to ensure data accuracy [31].

### Covariates

Data extracted from the NHANES database incorporates multitudinous covariates:(1) Demographics data including age, gender, race, education, marital status, ratio of family income to poverty (PIR). Educational level was divided into below high school, high school or equivalent, and college or above. Marital status was classified into three subgroups: married or living with a partner, never married, and widowed, divorced, or separated. PIR was divided into: ≤ 1.5, 1.5–4.0, and >4.0. (2) Examination information including blood pressure, weight, height, body mass index (BMI) and WC. BMI was divided into: < 25, 25- < 30, ≥ 30. The body measurement data were collected by trained health technicians at the mobile examination center. (3) Laboratory variables involving glycohemoglobin (HbA1c), fasting plasma glucose (FPG), total cholesterol (TC), triglyceride (TG), low density lipoprotein cholesterol (LDL-C), high density lipoprotein cholesterol (HDL-C), total protein (TP), alanine aminotransferase (ALT), aspartate aminotransferase (AST), alkaline phosphatase (ALP), gamma glutamyl transferase (GGT), lactate dehydrogenase (LDH) and other clinical indicators. (4) Questionnaire information including comorbidities, alcohol use, smoking, taking insulin now. Comorbidities (hypertension, diabetes) were identified by self-report, that is, “Have you/Has SP ever been told by a doctor or other health professional that you/s/he had hypertension/diabetes or borderline.” Smoking and alcohol consumption were measured by the question “Have you smoked at least 100 cigarettes in your life?” And “Have you consumed at least 12 alcoholic beverages of any type in any given year/lifetime?” (yes/no) to classify as active (positive) and non-active (non-positive). “Is SP/Are you now taking insulin” (yes/no) to classify as use and non-use.

### Statistical analysis

All analyses were performed using R statistical software (version 4.3.2) and were weighted according to sample weights from the National Center for Health Statistics to account for the complex, multistage probability sampling design. Baseline characteristics were presented in accordance with the quartiles of eGDR. Continuous variables were shown as mean±standard deviation (SD) for normally distributed data or median (Quartile 1, Quartile 3) for skew distributional data, and comparing different groups by analysis of variance (ANOVA) or Kruskal-Wallis H test when appropriate. Categorical variables were displayed as frequency and percentage, and the differences between groups were examined by chi-square tests. Receiver operating characteristic (ROC) curves were constructed, and Harrell’s C-index was applied to compare the predictive value of different cardiovascular disease-related indicators for cardiovascular disease, the combination of the indicators (model 1, 2, 3) with cardiovascular disease. ROC analysis was used to screen out the two best indicators for predicting CVD. Internal validation of the final model was performed using ROC analysis (grouping: 1999–2008, 2009–2018). Restricted cubic spline (RCS) was used to analyze the nonlinear relationship between the selected indicators and CVD, and threshold effect analysis was used to calculate the specific inflection information between the selected indicators and CVD. Three machine learning methods (SVM-RFE, XGBoost, and Boruta algorithms) were wielded to screen out important, relevant features. The top 20 features of each algorithm were ultimately combined to build a subset of covariates applied in Model 3. Logistic regression was performed to navigate the association between the combination of indicators and CVD. Model 1 adjusted for no covariates. Model 2 adjusted for age, gender, race, education, ratio of family income to poverty and marital status. Model 3 was adjusted according to the subset combining the top 20 most important variables obtained from three methods. Furthermore, we applied the net reclassification improvement (NRI), and integrated discrimination improvement (IDI) index to corroborate the accuracy and discriminative power of the models. Concurrently, we used a calibration curve to evaluate the consistency between predicted probability and actual occurrence rate. In addition, decision curve analysis (DCA) assesses the model’s clinical net benefit. Finally, subgroup analysis was performed to determine whether there were differences in the association between the combination of the indicators and CVD in different subgroups.

## Results

### Baseline characteristics

The baseline characteristics of the participants stratified by quartiles of the eGDR are shown in Table 1. The data revealed a significant age gradient decline with increasing eGDR values (Q1: 58.73 ± 14.82 years vs Q4: 38.46 ± 17.66 years, P < 0.001), accompanied by stepwise improvements in systolic blood pressure (133.40 ± 19.55 vs 114.59 ± 14.87 mmHg), WC (114.05 ± 13.94 vs 81.23 ± 6.40 cm), and glycemic control markers (FPG: 6.99 ± 2.67 vs 5.2 ± 0.58 mmol/L). Significant intergroup differences (P < 0.001 for all comparisons) were observed in gender distribution, racial composition, education level, and marital status. Notably, the Q4 group exhibited higher educational attainment (61.5% college or above) and superior socioeconomic status (29.7% with PIR > 4.0). In addition, median insulin levels decreased substantially from 86.28 pmol/L (Q1) to 36.36 pmol/L (Q4). The incidence rates of CVD and its subtypes, stroke were generally consistent with those in previous studies [32] (Table 1).

**Table 1:** Baseline characteristics of study individuals according to quartiles of the eGDR.

Variables	Overall (n = 14237)	eGDR Q1 < 5.84 (n = 3559)	eGDR Q2 5.84–8.61 (n = 3560)	eGDR Q3 8.61–10.11 (n = 3559)	eGDR Q4 > 10.11 (n = 3559)	*P* value
Age, years	48.98 ± 17.66	58.73 ± 14.82	52.86 ± 17.16	45.87 ± 16.21	38.46 ± 17.66	<0.001
Age>=60, years, n(%)	4581(32.2)	1940(54.5)	1400(39.3)	800(22.5)	441(12.4)	<0.001
Gender, n(%)						<0.001
Male	6985(49.1)	1904(53.5)	1712(48.1)	1982(55.7)	1387(39.0)	
Female	7252(50.9)	1655(46.5)	1848(51.9)	1577(44.3)	2172(61.0)	
Race, n(%)						<0.001
Mexican American	2394(16.8)	447(13.3)	661(17.2)	799(22.5)	510(14.3)	
Other Hispanic	1219(8.6)	286(8.0)	316(8.9)	343(9.6)	274(7.7)	
Non-Hispanic White	6663(46.8)	1713(48.1)	1695(47.6)	1593(44.8)	1662(46.7)	
Non-Hispanic Black	2661(18.7)	897(25.2)	670(18.8)	523(14.7)	571(16.0)	
Other Race	1300(9.1)	189(5.3)	268(7.5)	301(8.5)	542(15.2)	
Education level, n(%)						<0.001
Below high school	1426(10.0)	437(12.3)	390(11.0)	380(10.7)	219(6.2)	
High school or equivalent	5248(36.9)	1444(40.6)	1351(37.9)	1302(36.6)	1151(32.3)	
College or above	7563(53.1)	1678(47.1)	1819(51.1)	1877(52.7)	2189(61.5)	
Ratio of family income to poverty, n(%)						<0.001
<=1.5	4893(34.4)	1329(37.3)	1210(34.0)	1197(33.6)	1157(32.5)	
> 1.5-4.0	5484(38.5)	1368(38.4)	1427(40.1)	1344(37.8)	1345 (8,37)	
> 4.0	3860(27.1)	862(24.3)	923(25.9)	1018(28.6)	1057(29.7)	
Marital status						<0.001
Married or living with a partner	8895(62.5)	2202(61.9)	2262(63.5)	2396(67.3)	2035(57.2)	
Never married	2410(16.9)	365(10.3)	467(13.1)	537(15.1)	1041(29.2)	
Widowed, divorced, or separated	2932(20.6)	992(27.9)	831(23.3)	626(37.6)	483(13.6)	
Systolic blood pressure,mmHg	124.19 ± 19.02	133.40 ± 19.55	128.43 ± 19.64	120.36 ± 15.75	114.59 ± 14.87	<0.001
Diastolic blood pressure,mmHg	70.62 ± 11.51	72.77 ± 12.57	72.03 ± 11.89	70.34 ± 10.64	67.32 ± 10.01	<0.001
Weight, kg	81.45 ± 20.69	97.41 ± 22.12	85.19 ± 20.43	79.73 ± 10.80	63.49 ± 9.81	<0.001
Height, cm	167.66 ± 9.99	168.43 ± 10.20	167.29 ± 10.33	168.53 ± 9.76	166.37 ± 9.53	<0.001
Body mass index, kg/m2						<0.001
< 25	4249(29.8)	108(3.0)	793(22.3)	574(16.1)	2774(77.9)	
25- < 30	4859(34.1)	954(26.8)	1032(29.0)	2125(59.7)	748(21.0)	
>=30	5129(36.1)	2497(70.2)	1735(48.7)	860(24.2)	37(1.1)	
Waist circumference, cm	98.86 ± 16.06	114.05 ± 13.94	102.92 ± 14.41	97.25 ± 5.29	81.23 ± 6.40	<0.001
Glycohemoglobin(%)	5.68 ± 1.01	6.35 ± 1.49	5.72 ± 0.92	5.43 ± 0.46	5.21 ± 0.35	<0.001
Fasting plasma glucose, mmol/L	5.94 ± 1.77	6.99 ± 2.67	5.98 ± 1.62	5.56 ± 0.77	5.21 ± 0.58	<0.001
Insulin, pmol/L	57.54(37.17,91.38)	86,28(57.06,134.82)	66.66(42.30,102.95)	56.76(40.44,81.24)	36.36(25.74,51.54)	<0.001
Total cholesterol, mmol/L	5.01 ± 1.05	4.91 ± 1.07	5.11 ± 1.07	5.18 ± 1.04	4.83 ± 1.00	<0.001
Triglyceride, mmol/L	1.37 ± 0.76	1.56 ± 0.79	1.46 ± 0.78	1.42 ± 0.75	1.03 ± 0.58	<0.001
LDL-cholesterol, mmol/L	2.98 ± 0.92	2.91 ± 0.94	3.06 ± 0.93	3.17 ± 0.90	2.79 ± 0.85	<0.001
HDL-cholesterol, mmol/L	1.39 ± 0.41	1.28 ± 0.35	1.38 ± 0.42	1.36 ± 0.38	1.56 ± 0.43	<0.001
Sodium, mmol/L	139.24 ± 2.33	139.23 ± 2.51	139.34 ± 2.38	139.28 ± 2.16	139.12 ± 2.25	<0.001
Potassium, mmol/L	4.04 ± 0.35	4.06 ± 0.38	4.05 ± 0.36	4.04 ± 0.32	3.98 ± 0.32	<0.001
Chloride, mmol/L	103.47 ± 2.93	102.96 ± 3.21	103.50 ± 3.06	103.91 ± 2.66	103.52 ± 2.68	1
Calcium, mmol/L	2.34 ± 0.09	2.34 ± 0.09	2.34 ± 0.10	2.33 ± 0.09	2.33 ± 0.09	1
Phosphorus, mmol/L	1.17 ± 0.18	1.17 ± 0.18	1.17 ± 0.18	1.16 ± 0.17	1.20 ± 0.17	1
Iron, umol/L	16.09 ± 6.55	15.10 ± 5.85	15.64 ± 6.32	16.55 ± 6.51	17.08 ± 7.26	<0.001
Total protein, g/dL	7.18 ± 0.48	7.18 ± 0.49	7.16 ± 0.49	7.18 ± 0.48	7.21 ± 0.48	<0.001
Globulin, g/dL	2.97 ± 0.46	3.05 ± 0.48	2.99 ± 0.47	2.94 ± 0.43	2.88 ± 0.42	<0.001
Albumin, g/dL	4.21 ± 0.35	4.12 ± 0.33	4.17 ± 0.35	4.23 ± 0.35	4.33 ± 0.34	<0.001
ALT, U/L	21.00(16.00,28.00)	22.00(17.00,30.00)	21.00(16.00,30.00)	22.00(17.00,30.00)	18.00(14.00,23.00)	0
AST, U/L	22.00(19.00,27.00)	23.00(19.00,28.00)	23.00(19.00,27.00)	22.00(19.00,27.00)	21.00(18.00,25.00)	<0.001
Alkaline phosphotase, U/L	70.84 ± 26.02	75.23 ± 28.28	74.30 ± 27.40	70.52 ± 24.36	63.31 ± 21.87	<0.001
GGT, U/L	20.00(14.00,31.00)	24.00(17.00,37.00)	21.00(15.00,33.00)	20.00(14.00,31.00)	15.00(12.00,21.00)	<0.001
LDH, U/L	133.91 ± 34.54	140.10 ± 33.31	137.49 ± 41.52	132.24 ± 31.48	125.80 ± 28.78	<0.001
Bilirubin, umol/L	11.92 ± 5.26	11.62 ± 4.92	11.52 ± 5.14	12.03 ± 5.19	12.51 ± 5.71	<0.001
Uric acid, umol/L	325.28 ± 84.99	362.57 ± 88.24	334.12 ± 81.99	321.82 ± 77.23	282.61 ± 81.58	<0.001
Blood urea nitrogen, mmol/L	4.82 ± 2.03	5.47 ± 2.56	4.95 ± 2.14	4.57 ± 1.58	4.27 ± 1.43	<0.001
Creatinine, umol/L	77.73 ± 36.75	84.41 ± 44.88	80.16 ± 49.56	75.27 ± 22.70	71.10 ± 17.82	<0.001
Alcohol use, n(%)						<0.001
Non-Active alcohol user	3649(25.6)	1012(28.4)	965(27.1)	829(23.3)	843(23.7)	
Active alcohol user	10588(74.4)	2547(71.6)	2595(72.9)	2730(76.7)	2716(74.4)	
Smoking, n(%)						<0.001
Non-Active smoker	7698(54.1)	1716(48.2)	1867(52.4)	1972(55.4)	2143(60.2)	
Active smoker	6539(45.9)	1843(51.8)	1693(47.6)	1587(44.6)	1416(39.8)	
Diabetes, n(%)						<0.001
No	12357(86.8)	2350(66.0)	3093(86.9)	3398(95.5)	3516(98.8)	
Yes	1591(11.2)	1065(29.9)	374(10.5)	123(3.5)	29(0.8)	
Borderline	289(2.0)	133(4.0)	93(2.6)	38(1.1)	14(0.4)	
Taking insulin now, n(%)						<0.001
No	13839(97.2)	3261(91.6)	3475(97.6)	3549(99.7)	3554(99.9)	
Yes	398(2.8)	298(8.4)	85(2.4)	10(0.3)	5(0.1)	
Hypertension, n(%)						<0.001
No	9375(65.8)	246(6.9)	2022(56.8)	3548(99.7)	3551(99.8)	
Yes	4862(34.2)	3313(93.1)	1538(43.2)	11(0.3)	8(0.2)	
Cardiovascular disease, n(%)	1259(8.8)	700(19.7)	367(10.3)	125(3.5)	67(1.9)	<0.001
Congestive heart failure, n(%)	387(2.7)	239(6.7)	98(2.8)	34(1.0)	16(0.4)	<0.001
Coronary artery disease, n(%)	544(3.8)	304(8.5)	169(4.7)	48(1.3)	23(0.6)	<0.001
Angina, n(%)	363(2.5)	209(5.9)	95(2.7)	37(1.0)	22(0.6)	<0.001
Stroke, n%	467(3.3)	248(7.0)	151(4.2)	42(1.2)	26(0.7)	<0.001
ABSI, mean±SE	0.0815 ± 0.0049	0.0839 ± 0.0046	0.0824 ± 0.0047	0.0814 ± 0.0043	0.0784 ± 0.0043	<0.001

### ROC curve analysis of cardiovascular disease-related indicators

**Fig 2:**
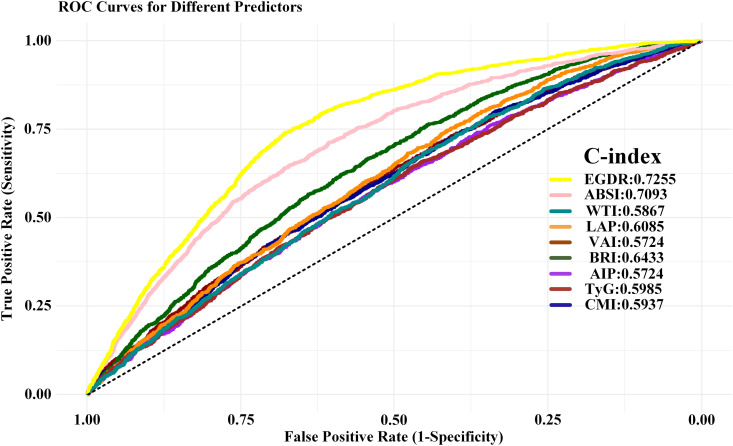
Receiver operating characteristic curves for cardiovascular disease-related indicators. Abbreviations: eGDR, estimated glucose disposal rate; ABSI, a body shape index; WTI, waist triglyceride index; LAP, lipid accumulation product; VAI, visceral adiposity index; BRI, body roundness index; AIP, atherogenic index of plasma; TyG, triglyceride glucose; CMI, cardiometabolic index.

The predictive performance of eGDR and ABSI was significantly greater than that of the other indices (Fig 2). Among the assessed predictors, eGDR exhibited the highest Harrell’s C-index (0.7255), followed closely by ABSI (0.7093), indicating superior discriminatory power for cardiovascular disease risk. In contrast, the C-index of the other indices was lower, including BRI (0.6433), LAP (0.6085), and TyG (0.5985), suggesting moderate predictive capabilities. Notably, VAI and AIP demonstrated the lowest C-indices (both 0.5724), indicating limited prognostic value. Furthermore, ROC curve analysis confirmed that eGDR and ABSI outperformed other body composition and metabolic indices in predicting cardiovascular risk. These findings underscore the robustness of eGDR and ABSI as reliable markers for cardiovascular disease, outperforming traditional lipid and adiposity-related indicators.

Given the results above, we sought to explore the potential additive effect of the eGDR and ABSI, which incorporate different parameters in their formulas, on CVD.

### RCS curve and threshold analysis of eGDR and ABSI with cardiovascular disease

**Fig 3:**
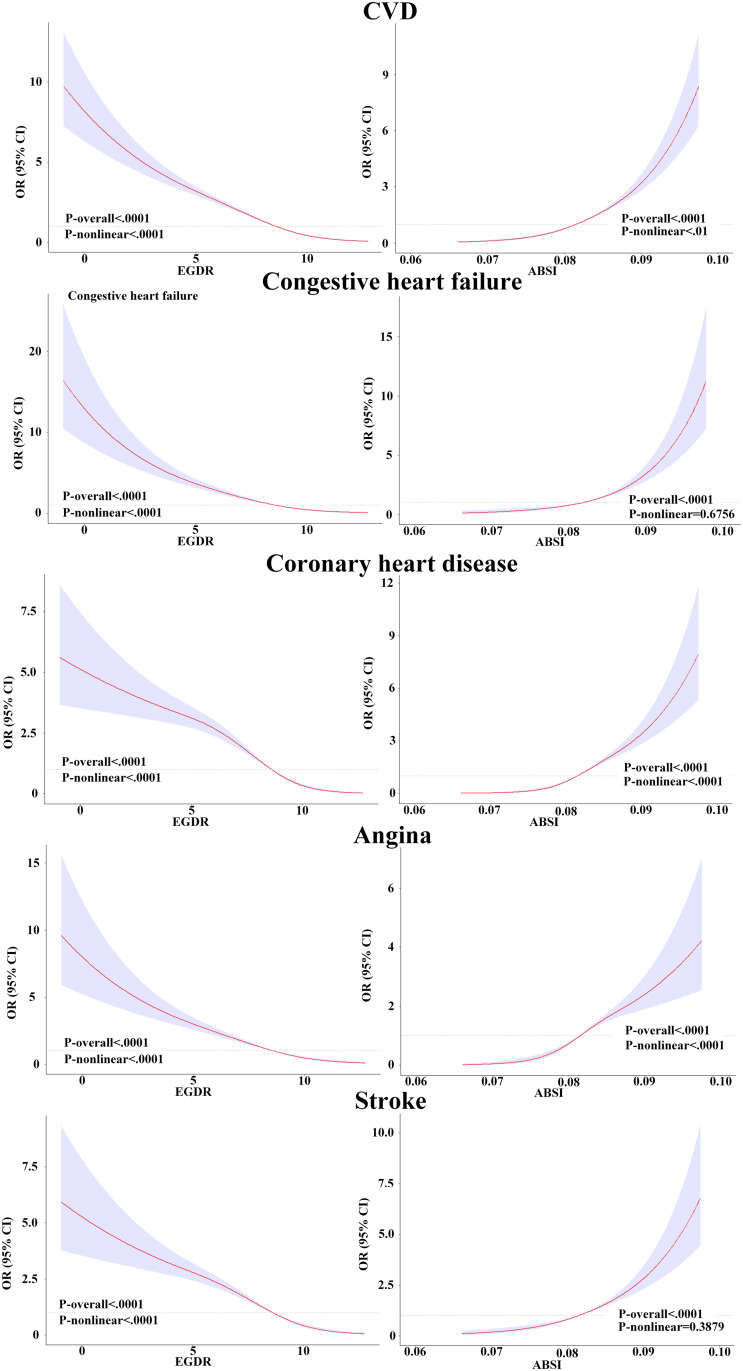
Restricted cubic spline plots analysis of eGDR and ABSI with cardiovascular disease.

The RCS analysis revealed a significant nonlinear relationship between eGDR, ABSI, and CVD risk (Fig 3). Overall, eGDR exhibited an inverse relationship with CVD. In congestive heart failure, the odds ratio (OR) sharply declined from approximately 16 at an eGDR of 0 to around 6 at an eGDR of 5, after which it stabilized. Similarly, for coronary heart disease, the OR decreased from nearly 6 at an eGDR of 0 to approximately 0.5 at an eGDR of 10. In contrast, ABSI showed a strong positive correlation with CVD risk. For coronary heart disease, the OR increased from about 0.1 at an ABSI of 0.07 to nearly 8 at an ABSI of 0.10.

Threshold analysis further confirmed the nonlinear associations and inflection points between eGDR, ABSI, and CVD risk (Table 2). A significant inflection point was identified at 6.448 ± 1.383 (p < 0.001) for eGDR, below which the risk of CVD increased significantly, while the protective effect stabilized beyond this threshold. For specific conditions include stroke, the eGDR threshold was 6.685 ± 1.145 (p < 0.001), indicating a strong protective effect above this value. Conversely, for ABSI, a critical threshold was found at 0.086 ± 0.002 (p < 0.001), beyond which the risk of CVD increased sharply. These thresholds represent critical turning points in the risk trajectory and provide interpretable reference points for categorizing risk groups. Additionally, as this study primarily focuses on overall CVD, we selected the CVD break-point as the primary reference for analysis.

**Table 2:** Threshold analysis of eGDR and ABSI with cardiovascular disease.

Variables	EGDR			ABSI		
	Break-point	Effect		Break-point	Effect	
		Estimate	*P* value		Estimate	*P* value
CVD	6.448 ± 1.383	−0.2159 ± 0.0474	<0.001	0.086 ± 0.002	88.96 ± 34.91	0.0108
Congestive heart failure	3.709 ± 1.416	−0.4403 ± 0.1276	<0.001	0.075 ± 0.003	95.35 ± 153.4	0.5341
Coronary heart disease	7.557 ± 0.730	−0.1563 ± 0.05944	0.008	0.086 ± 0.001	34.84 ± 6.236	0.0064
Angina	11.068 ± 0.213	−0.2662 ± 0.04065	<0.001	0.078 ± 0.001	261.9 ± 163.4	0.1088
Stroke	6.685 ± 1.145	−0.1511 ± 0.06878	0.0281	0.080 ± 0.005	58.52 ± 67.87	0.3885

### Feature selection and association between the combination of the eGDR and ABSI with cardiovascular disease

**Fig 4:**
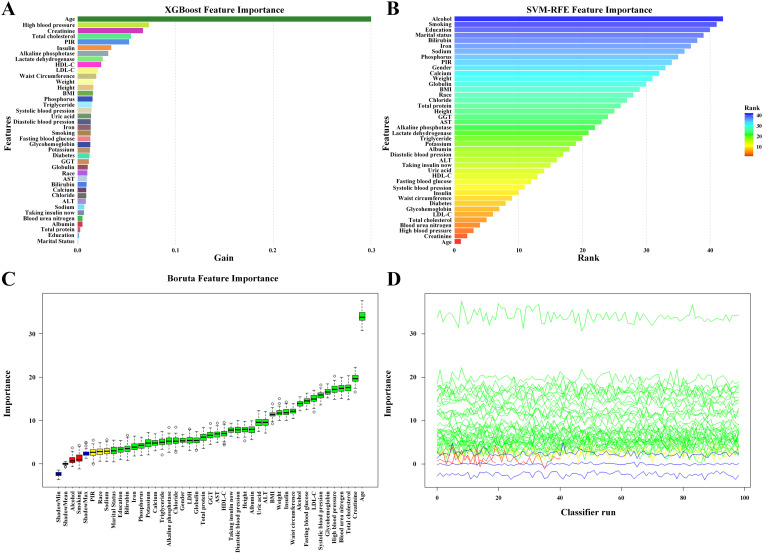
Feature selection results from machine learning algorithms: XGBoost (A), SVM-RFE (B), and Boruta (C, D). **(A)** Top 20 features ranked by importance in the XGBoost model; gain longer indicate higher importance. **(B)** Final 20 features eliminated by the SVM-RFE method; top ranks indicate later removal. **(C)** Feature importance from Boruta after 500 iterations. The x-axis shows input variables; the y-axis represents importance scores (Z-scores). Dark blue boxes indicate the distribution (min, mean, max) of shadow features, while green, yellow, and red boxes represent confirmed, tentative, and rejected features, respectively. **(D)** Iterative selection process in the Boruta algorithm.

To examine the link between combined eGDR and ABSI and cardiovascular disease prevalence, three logistic regression models were developed. Model 3 adjustments were based on variables selected via SVM-RFE, XGBoost, and Boruta algorithms (Fig 4 and S1 Table), including: age, creatinine, high blood pressure, blood urea nitrogen, total cholesterol, LDL-cholesterol, glycohemoglobin, diabetes, waist circumference, insulin, systolic blood pression, fasting blood glucose, HDL-cholesterol, uric acid, taking insulin now, ALT, diastolic blood pression, albumin, potassium, triglyceride.

According to logistic regression results, higher eGDR and lower ABSI were significantly linked to increased odds of CVD (Table 3). When analyzing the combination of eGDR and ABSI as a categorical variable in an unadjusted model, a demographic-adjusted model, and a machine learning-adjusted model, the results demonstrated a significant impact of the combination on CVD risk: for the low eGDR and high ABSI group compared to the reference group, the ORs were 11.792 (11.772, 11.812) in Model 1, 3.190 (3.184, 3.197) in Model 2, and 1.323 (1.319, 1.327) in Model 3, all with p < 0.001. Similarly, the association analyses for congestive heart failure, coronary heart disease, angina, and stroke showed strong correlations in Model 1, while Model 3 also indicated a certain degree of association, with ORs of 1.357 (1.350, 1.364), 1.287 (1.281, 1.292), 1.249 (1.242, 1.255), and 1.010 (1.006, 1.015), all with p < 0.05.

**Table 3:** Association of the combination of the eGDR and ABSI with cardiovascular disease.

Models	Group				*P* for trend
	High eGDER and low ABSI (eGDR > 6.448 and ABSI<0.086)	Low eGDER and low ABSI (eGDR<=6.448 and ABSI<0.086)	High eGDER and high ABSI (eGDR > 6.448 and ABSI>=0.086)	Low eGDER and high ABSI (eGDR<=6.448 and ABSI>=0.086)	
Cardiovascular disease					<0.001
Model 1	Reference	5.131(5.123,5.139) <0.001	4.211(4.202,4.219) <0.001	11.792(11.772,11.812) <0.001	
Model 2	Reference	2.864(2.859,2.868) <0.001	1.501(1.498,1.505) <0.001	3.190(3.184,3.197) <0.001	
Model 3	Reference	1.189(1.186,1.193) <0.001	1.546(1.543,1.550) <0.001	1.323(1.319,1.327) <0.001	
Congestive heart failure					<0.001
Model 1	Reference	5.017(5.003,5.032) <0.001	4.316(4.299,4.333) <0.001	14.169(14.128,14.211) <0.001	
Model 2	Reference	2.786(2.778,2.795) <0.001	1.632(1.625,1.639) <0.001	3.996(3.983,4.009) <0.001	
Model 3	Reference	1.874(1.869,1.883) <0.001	1.787(1.780,1.795) <0.001	1.357(1.350,1.364) <0.001	
Coronary heart disease					<0.001
Model 1	Reference	5.131(5.120,5.143) <0.001	4.568(4.554,4.582) <0.001	13.067(13.036,13.098) <0.001	
Model 2	Reference	2.661(2.665,2.668) <0.001	1.410(1.406,1.415) <0.001	3.026(3.018,3.034) <0.001	
Model 3	Reference	1.166(1.161,1.170) <0.001	1.482(1.477,1.489) <0.001	1.287(1.281,1.292) <0.001	
Angina					<0.001
Model 1	Reference	5.091(5.078,5.104) <0.001	3.473(3.460,3.487) <0.001	10.591(10.561,10.620) <0.001	
Model 2	Reference	1.128(1.125,1.132) <0.001	1.162(1.151,1.175) <0.001	1.162(1.158,1.166) <0.001	
Model 3	Reference	1.130(1.125,1.136) <0.001	1.416(1.410,1.422) <0.001	1.249(1.242,1.255) <0.001	
Stroke					<0.001
Model 1	Reference	4.131(4.121,4.141) <0.001	3.021(3.009,3.031) <0.001	7.581(7.561,7.602) <0.001	
Model 2	Reference	2.220(2.214,2.225) <0.001	1.132(1.128,1.136) <0.001	2.174(2.168,2.181) <0.001	
Model 3	Reference	1.028(1.024,1.032) 0.023	1.192(1.187,1.196) <0.001	1.010(1.006,1.015) 0.045	

Model 1: Unadjusted.

Model 2: adjusted for age, gender, race, education, ratio of family income to poverty, marital status.

Model 3: adjusted for age, creatinine, high blood pressure, blood urea nitrogen, total cholesterol, LDL-cholesterol, glycohemoglobin, diabetes, waist circumference, insulin, systolic blood pression, fasting blood glucose, HDL-cholesterol, uric acid, taking insulin now, ALT, diastolic blood pression, albumin, potassium, triglyceride.

### Model performance evaluation

**Fig 5:**
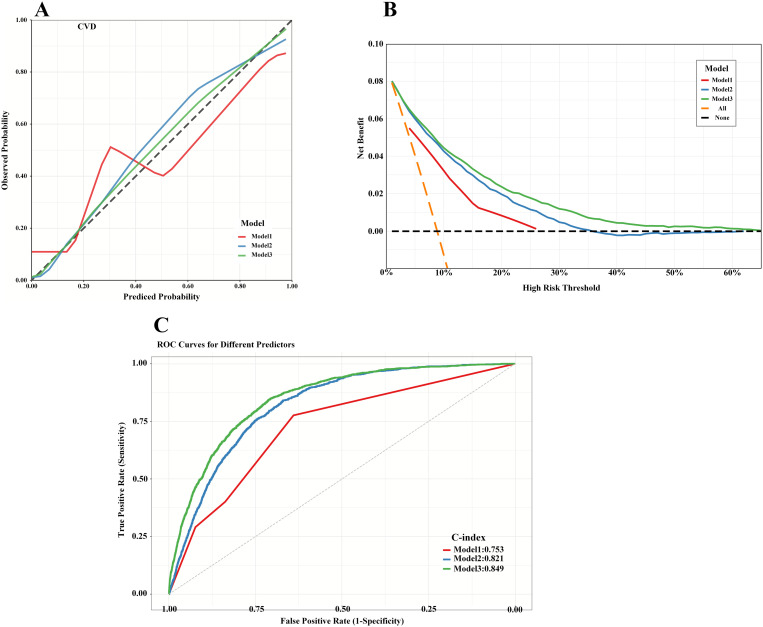
Model performance assessment. **(A)** Calibration curves: The diagonal line indicates perfect agreement between predicted and observed probabilities. The three curves represent bootstrapped calibration lines for Models 1-3. **(B)** Decision curve analysis illustrating the net benefit of each model in predicting cardiovascular disease. **(C)** ROC curves of the logistic regression models assessing the association between combined eGDR and ABSI and cardiovascular disease.

The ROC curve showed that the combination of the eGDR and ABSI in the model adjusted by machine learning had a significant effect on CVD (C-statistics: model 3 0.849 and model 1 0.753) had higher accuracy and better discrimination (Fig 5 C, Table 4). The results of internal validation remained consistent (S1 Fig). DeLong test showed that the difference between the AUCs of model 1 and model 2, model 3 was statistically significant (p < 0.05). Through calibration curve and decision curve analysis (Fig 5 A, B), model 3 was more reliable than model2 and model1 in evaluating CVD, with stronger agreement between predicted and observed probabilities. And higher model net benefit. Furthermore, NRI and IDI analyses demonstrated that the machine learning–adjusted model outperformed the baseline model, with all p-values < 0.05 (Table 4). Overall, Model 3 exhibited superior calibration, discrimination, and clinical utility compared to the other models.

**Table 4:** Model performance evaluation of different models. NRI net reclassification improvement, IDI integrated discrimination improvement.

NRI	CVD	Congestive heart failure	Coronary heart disease	Angina	Stroke
	Estimate (95% CI) *P* value				
Model 1	Reference	Reference	Reference	Reference	Reference
Model 2	0.014(0.008,0.021) <0.001	0.034(0.025,0.041) <0.001	0.003(0.001,0.005) <0.001	0.015(0.007,0.024) <0.001	0.009(0.002,0.015) <0.001
Model 3	0.108(0.092,0.125) <0.001	0.049(0.027,0.073) <0.001	0.015(0.005,0.025) <0.001	0.018(0.011,0.027) <0.001	0.021(0.006,0.046) <0.001
Model 3 VS 2	0.093(0.077,0.110) <0.001	0.017(0.009,0.023) <0.001	0.013(0.004,0.025) <0.001	0.004(0.001,0.010) <0.001	0.011(0.005,0.020) <0.001
**IDI**					
Model 1	Reference	Reference	Reference	Reference	Reference
Model 2					
Total	0.063(0.058,0.069) <0.001	0.023(0.017,0.026) <0.001	0.053(0.046,0.060) <0.001	0.018(0.014,0.022) <0.001	0.027(0.023,0.031) <0.001
Event	0.058(0.052,0.063) <0.001	0.022(0.018,0.026) <0.001	0.051(0.045,0.058) <0.001	0.017(0.014,0.021) <0.001	0.026(0.022,0.030) <0.001
Non-event	0.006(0.005,0.007) <0.001	0.002(0.001,0.003) 0.004	0.002(0.001,0.003) 0.003	0.001(0.000,0.001) 0.045	0.001(0.000,0.001) 0.040
Model 3					
Total	0.107(0.097,0.115) <0.001	0.077(0.062,0.091) <0.001	0.084(0.075,0.094) <0.001	0.033(0.027,0.040) <0.001	0.034(0.029,0.039) <0.001
Event	0.097(0.088,0.105) <0.001	0.074(0.060,0.089) <0.001	0.081(0.071,0.090) <0.001	0.032(0.027,0.039) <0.001	0.033(0.028,0.038) <0.001
Non-event	0.009(0.008,0.011) <0.001	0.004(0.001,0.005) <0.001	0.003(0.002,0.004) 0.002	0.001(0.000,0.001) 0.042	0.001(0.001,0.002) 0.024
Model 3 VS 2					
Total	0.043(0.036,0.050) <0.001	0.054(0.041,0.069) <0.001	0.031(0.023,0.039) <0.001	0.016(0.011,0.021) <0.001	0.007(0.003,0.012) <0.001
Event	0.039(0.032,0.046) <0.001	0.053(0.039,0.067) <0.001	0.030(0.022,0.038) <0.001	0.015(0.010,0.021) <0.001	0.007(0.002,0.012) <0.001
Non-event	0.004(0.003,0.005) <0.001	0.001(0.001,0.002) 0.020	0.002(0.001,0.003) 0.003	0.000(0.000,0.001) 0.046	0.000(−0.002,0.001) 0.857

### Subgroup analysis of the association between the combination of the eGDR and ABSI with cardiovascular disease

**Fig 6:**
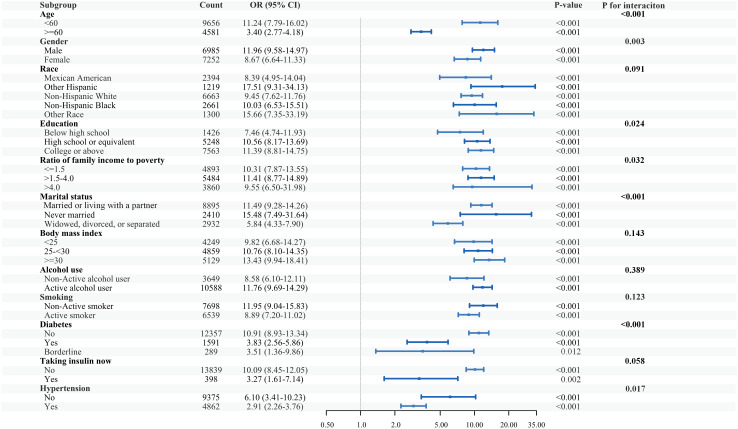
Subgroup analysis of the association between the combination of the eGDR and ABSI with cardiovascular disease.

Subgroup analyses were performed across categories defined by age, gender, race, education, PIR, marital status, BMI, alcohol, smoking, diabetes, current insulin use, and hypertension. For CVD, the results of all subgroups remained consistent with the previous outcomes (Fig 6). Interaction effect analyses unveiled the association of the combination of the eGDR and ABSI with CVD was more pronounced (p for interaction <0.001) in the population with younger age (<60) than older age (≥60). Meanwhile, the male, high educational level, low PIR, never married posed a noticeable leverage on the association (p for interaction <0.05).

## Discussion

In this study, we explored the association between eGDR, ABSI, and CVD using the NHANES database. The main findings of this study are summarized as follows: [1] The relationship between eGDR and CVD exhibits an “L”-shaped pattern, while the relationship between ABSI and CVD follows a “J”-shaped pattern. [2] After adjusting for key and relevant variables identified through a machine learning algorithm, which differs from traditional clinical selection methods, we found that eGDR and ABSI have a potential additive effect on CVD risk. Specifically, the combination of eGDR ≤ 6.448 and ABSI ≥0.086 can effectively identify individuals at risk for CVD in the population. [3] Further evaluation of model performance confirmed that the machine learning-adjusted model demonstrated better accuracy and discriminative ability compared to the baseline model. Our findings suggest that the combination of eGDR and ABSI may help improve CVD risk stratification in the population.

The eGDR, calculated with the use of a formula that incorporates laboratory and physical examination data, was originally developed to assess insulin resistance in patients with type 1 diabetes [30]. Recent studies have expanded its application to predict diabetes-related complications and CVD risk assessment [12]. A retrospective study of patients with type 2 diabetes found that lower eGDR was a predictor of worsening renal function in patients with type 2 diabetes [33]. In a 10-year community-based cohort study of community-dwelling older adults, eGDR was an independent predictor of all-cause mortality and was mediated by risk of arterial stiffness [34]. Penno et al. found a significant association between eGDR and all-cause mortality in follow-up patients with type 2 diabetes and this association was stronger in men and younger people [35].

The ABSI, calculated adjusted for waist circumference, BMI, and height, was introduced to more accurately quantify health risks associated with central obesity than conventional BMI, which does not account for fat distribution, and ABSI has been shown to be a stronger predictor of obesity-related morbidity and mortality than BMI alone [23]. In addition, the ABSI was also found to be directly associated with all-cause mortality (HR = 1.43, 95%CI: 1.07–1.92) [36]. A retrospective cross-sectional study of 46,872 residents in Japan found a positive association between ABSI and arteriosclerosis index [37]. Zhang et al. found that ABSI was an independent predictor of stroke (HR = 1.33, 95%CI: 1.06–1.68) in a retrospective study of 8,257 individuals aged 45 years and older [38].

Previous studies have separately emphasized the relationship between eGDR or ABSI with the risk of CVD and its subtypes [11,13]. Our findings also confirmed that both eGDR and ABSI are significant predictors of CVD risk. Specifically, eGDR showed a negative correlation with CVD, with a threshold of 6.448, while ABSI exhibited a positive correlation, with a threshold of 0.086. These thresholds represent critical turning points in the risk trajectory and provide interpretable reference points for categorizing risk groups. While these values may be influenced by sample-specific characteristics, the use of a nationally representative NHANES dataset supports their robustness [39]. This dual association highlights the potential additive effect of eGDR and ABSI in predicting CVD. In addition, the results of ROC analysis showed that the final model had a strong predictive value (C-statistics: 0.849), which was corresponding to the previous study on eGDR and CVD in the diabetic population (C-statistics: 0.814) [13]. This suggests that there is indeed some additive effect. However, in contrast to prior research [13,16], we found that the combined effect of eGDR and ABSI on cardiovascular disease was more pronounced in individuals under 60 years of age, suggesting that the concurrent adverse impact of metabolic and body composition factors may lead to extremely high cardiovascular risk in younger populations (OR = 11.24, 5%CI: 7.79–16.02). Additionally, the impact of eGDR and ABSI on cardiovascular disease risk was more significant among males, individuals with higher education levels, low-income groups, and unmarried individuals (P for interaction <0.05). Notably, when we applied a machine-learning-adjusted logistic regression model, the predictive performance of eGDR and ABSI for CVD improved significantly. This enhancement may be attributed to the integration of the top 20 variables contributing most to the outcome in the machine learning model, as well as the complementary information provided by the two biomarkers in different aspects of metabolic and body composition health, thereby ensuring sufficient predictive performance for the outcome variable. Meanwhile, compared to traditional tools like the Framingham Risk Score (FRS), which rely on age, sex, cholesterol, and smoking status [40], our model integrating eGDR and ABSI adds predictive value by reflecting insulin resistance and body composition. While FRS is widely used, it may underestimate risk in younger or metabolically unhealthy individuals with normal lipid levels. In contrast, our model showed stronger predictive performance (C-statistic: 0.849) and may better identify subclinical risk in metabolically unhealthy population [41].

Both eGDR and ABSI are influenced by central obesity (increased waist circumference), dyslipidemia, and hypertension, which are common components of CVD and are incorporated into their respective formulas [42]. However, the comprehensive impact of these factors on cardiovascular risk, particularly their combined effect, has not been fully explored. Previous studies have indicated that eGDR primarily reflects systemic insulin resistance, which plays a crucial role in the development of atherosclerosis through pathways including endothelial dysfunction, chronic low-grade inflammation, oxidative stress, and impaired lipid metabolism [43]. Insulin resistance reduces nitric oxide (NO) bioavailability, promoting vasoconstriction and vascular stiffness, while also enhancing the production of pro-inflammatory cytokines, including TNF-α and IL-6, further accelerating vascular damage [44]. In addition, insulin resistance contributes to dyslipidemia, characterized by elevated triglycerides, reduced HDL-cholesterol, and the formation of small, dense LDL particles, all of which are closely associated with atherosclerotic plaque progression [45]. On the other hand, ABSI reflects central obesity, which serves as a major source of adipokines and inflammatory mediators that exacerbate insulin resistance and vascular dysfunction [46,47]. Visceral adipose tissue releases excess free fatty acids, inducing lipotoxicity in endothelial cells and cardiomyocytes, thereby increasing the risk of cardiovascular events [48]. Moreover, visceral obesity is strongly associated with the overactivation of the renin-angiotensin-aldosterone system (RAAS), further promoting hypertension and endothelial dysfunction [49,50]. Taken together, eGDR and ABSI represent two interconnected yet distinct pathways that accelerate atherosclerosis and elevate CVD risk. This dual-channel effect on metabolic dysfunction and body composition may explain the higher observed ORs for CVD when both eGDR and ABSI are unfavorable. However, the combined effect of eGDR and ABSI reflects complex metabolic and physiological interactions, and the specific mechanisms underlying their detrimental cardiovascular outcomes require further investigation to be fully elucidated.

Since both eGDR and ABSI are independent prognostic predictors beyond traditional cardiovascular risk factors, our findings highlight the importance of considering both metabolic and body composition indicators when assessing cardiovascular risk, particularly in populations at high risk of CVD. Importantly, both eGDR and ABSI are derived from routinely available clinical data (HbA1c, blood pressure, waist circumference, etc.), making them feasible for implementation in primary care settings. Their combined use can potentially aid in early detection of individuals at high cardiovascular risk, especially in resource-limited settings where laboratory-based lipid profiles or imaging may not be readily available. After conducting clinical empirical studies to confirm its effectiveness, the next step is to develop simplified nomograms or online calculators to improve their clinical value.

The strengths of our study are as follows. First, the NHANES database has nationally representative samples with standardized measures and extensive demographic and clinical data, which reduces potential bias. Second, we simultaneously evaluated the combined effects of eGDR and ABSI on CVD risk, providing new insights into the complementary roles of both in cardiovascular pathophysiology. Third, we applied a variety of machine learning algorithms for model tuning, which improved the accuracy and predictive power of the model. Fourth, multiple advanced statistical methods and visualization techniques were applied to comprehensively evaluate the relationship between our model and CVD, including ROC, RCS analysis, interaction, NRI, IDI, calibration curve, and DCA curve. However, several limitations should be acknowledged. First, because the NHANES is a cross-sectional survey, a causal relationship between eGDR, ABSI, and CVD cannot be definitively determined. Second, although eGDR and ABSI incorporate key metabolic and anthropometric measures, they do not directly measure insulin resistance or central obesity by means of gold-standard techniques (hyperinsulinemic-euglycemic clamp testing or imaging-based assessment of fat distribution) and, further external validation is needed to confirm the generalizability and clinical applicability of these thresholds. Finally, although machine-learning techniques improve model performance, external validation in independent cohorts is needed to confirm the clinical applicability of our prediction model.

## Conclusions

The combined assessment of eGDR and ABSI can provide a more comprehensive cardiovascular risk assessment that goes beyond traditional risk factors. Compared with the unadjusted model and the population-adjusted model, the model constructed by the variables selected by the machine learning algorithm had better predictive performance for CVD. Given that both measures are derived from routine clinical measures, they provide a cost-effective and accessible approach to improving cardiovascular risk assessment in a large population.

## Supporting information

S1 Table Feature importance. (XLSX)

S1 Fig ROC internal validation. (TIF)

## References

[1] Chong B, Jayabaskaran J, Jauhari SM, Chan SP, Goh R, Kueh MTW, et al. Global burden of cardiovascular diseases: projections from 2025 to 2050. Eur J Prev Cardiol. 2024;zwae281. doi: 10.1093/eurjpc/zwae281 39270739

[2] Devesa A, Ibanez B, Malick WA, Tinuoye EO, Bustamante J, Peyra C, et al. Primary Prevention of Subclinical Atherosclerosis in Young Adults: JACC Review Topic of the Week. J Am Coll Cardiol. 2023;82(22):2152–62. doi: 10.1016/j.jacc.2023.09.817 37993206

[3] Zhang J, Tong H, Jiang L, Zhang Y, Hu J. Trends and disparities in China’s cardiovascular disease burden from 1990 to 2019. Nutr Metab Cardiovasc Dis. 2023;33(12):2344–54. doi: 10.1016/j.numecd.2023.07.039 37596135

[4] Roth GA, Mensah GA, Johnson CO, Addolorato G, Ammirati E, Baddour LM, et al. Global burden of cardiovascular diseases and risk factors, 1990-2019: update from the GBD 2019 study. J Am Coll Cardiol. 2020;76(25):2982–3021. 33309175 10.1016/j.jacc.2020.11.010 PMC7755038

[5] Wang H, Yu X, Guo J, Ma S, Liu Y, Hu Y, et al. Burden of cardiovascular disease among the Western Pacific region and its association with human resources for health, 1990-2021: a systematic analysis of the Global Burden of Disease Study 2021. Lancet Reg Health West Pac. 2024;51:101195. doi: 10.1016/j.lanwpc.2024.101195 39286450 PMC11404088

[6] Teo KK, Rafiq T. Cardiovascular Risk Factors and Prevention: A Perspective From Developing Countries. Can J Cardiol. 2021;37(5):733–43. doi: 10.1016/j.cjca.2021.02.009 33610690

[7] Gao Y, Wang M, Wang R, Jiang J, Hu Y, Wang W, et al. The predictive value of the hs-CRP/HDL-C ratio, an inflammation-lipid composite marker, for cardiovascular disease in middle-aged and elderly people: evidence from a large national cohort study. Lipids Health Dis. 2024;23(1):66. doi: 10.1186/s12944-024-02055-7 38429790 PMC10908181

[8] Damen JAAG, Hooft L, Schuit E, Debray TPA, Collins GS, Tzoulaki I, et al. Prediction models for cardiovascular disease risk in the general population: systematic review. BMJ. 2016;353:i2416. doi: 10.1136/bmj.i2416 27184143 PMC4868251

[9] Zhiting G, Jiaying T, Haiying H, Yuping Z, Qunfei Y, Jingfen J. Cardiovascular disease risk prediction models in the Chinese population- a systematic review and meta-analysis. BMC Public Health. 2022;22(1):1608. doi: 10.1186/s12889-022-13995-z 35999550 PMC9400257

[10] Xiao S, Wang X, Zhang G, Tong M, Chen J, Zhou Y, et al. Association of Systemic Immune Inflammation Index with Estimated Pulse Wave Velocity, Atherogenic Index of Plasma, Triglyceride-Glucose Index, and Cardiovascular Disease: A Large Cross-Sectional Study. Mediat Inflamm. 2023;2023:1966680. 10.1155/2023/1966680 PMC9946741 36846196

[11] Li Y, Zeng L. Comparison of seven anthropometric indexes to predict hypertension plus hyperuricemia among U.S. adults. Front Endocrinol (Lausanne). 2024;15:1301543. doi: 10.3389/fendo.2024.1301543 38524637 PMC10958198

[12] Zhang Z, Chen X, Jiang N. The triglyceride glucose related index is an indicator of Sarcopenia. Sci Rep. 2024;14(1):24126. doi: 10.1038/s41598-024-75873-5 39406884 PMC11480318

[13] Liao J, Wang L, Duan L, Gong F, Zhu H, Pan H, et al. Association between estimated glucose disposal rate and cardiovascular diseases in patients with diabetes or prediabetes: a cross-sectional study. Cardiovasc Diabetol. 2025;24(1):13. doi: 10.1186/s12933-024-02570-y 39806389 PMC11730478

[14] Zhang Y, Wang F, Tang J, Shen L, He J, Chen Y. Association of triglyceride glucose-related parameters with all-cause mortality and cardiovascular disease in NAFLD patients: NHANES 1999-2018. Cardiovasc Diabetol. 2024;23(1):262. doi: 10.1186/s12933-024-02354-4 39026233 PMC11264797

[15] Huang H, Xiong Y, Zhou J, Tang Y, Chen F, Li G, et al. The predictive value of estimated glucose disposal rate and its association with myocardial infarction, heart failure, atrial fibrillation and ischemic stroke. Diabetes Obes Metab. 2025;27(3):1359–68. doi: 10.1111/dom.16132 39743837

[16] Yan L, Zhou Z, Wu X, Qiu Y, Liu Z, Luo L, et al. Association between the changes in the estimated glucose disposal rate and new-onset cardiovascular disease in middle-aged and elderly individuals: A nationwide prospective cohort study in China. Diabetes Obes Metab. 2025;27(4):1859–67. doi: 10.1111/dom.16179 39762991 PMC11885094

[17] Tao L-C, Xu J-N, Wang T-T, Hua F, Li J-J. Triglyceride-glucose index as a marker in cardiovascular diseases: landscape and limitations. Cardiovasc Diabetol. 2022;21(1):68. doi: 10.1186/s12933-022-01511-x 35524263 PMC9078015

[18] Fahami M, Hojati A, Farhangi MA. Body shape index (ABSI), body roundness index (BRI) and risk factors of metabolic syndrome among overweight and obese adults: a cross-sectional study. BMC Endocr Disord. 2024;24(1):230. doi: 10.1186/s12902-024-01763-6 39468529 PMC11514815

[19] Yang Y, Li S, Ren Q, Qiu Y, Pan M, Liu G, et al. The interaction between triglyceride-glucose index and visceral adiposity in cardiovascular disease risk: findings from a nationwide Chinese cohort. Cardiovasc Diabetol. 2024;23(1):427. doi: 10.1186/s12933-024-02518-2 39604987 PMC11603997

[20] Liao F-J, Shen S-L, Bao H-L, Li H, Zhao Q-W, Chen L, et al. Identification and experimental validation of KMO as a critical immune-associated mitochondrial gene in unstable atherosclerotic plaque. J Transl Med. 2024;22(1):668. doi: 10.1186/s12967-024-05464-5 39026250 PMC11256392

[21] Lee Y, Cappellato M, Di Camillo B. Machine learning-based feature selection to search stable microbial biomarkers: application to inflammatory bowel disease. Gigascience. 2022;12:giad083. doi: 10.1093/gigascience/giad083 37882604 PMC10600917

[22] Sun Q, Ren Q, Du L, Chen S, Wu S, Zhang B, et al. Cardiometabolic Index (CMI), Lipid Accumulation Products (LAP), Waist Triglyceride Index (WTI) and the risk of acute pancreatitis: a prospective study in adults of North China. Lipids Health Dis. 2023;22(1):190. doi: 10.1186/s12944-023-01948-3 37946249 PMC10633920

[23] Krakauer NY, Krakauer JC. A new body shape index predicts mortality hazard independently of body mass index. PLoS One. 2012;7(7):e39504. doi: 10.1371/journal.pone.0039504 22815707 PMC3399847

[24] Thomas DM, Bredlau C, Bosy-Westphal A, Mueller M, Shen W, Gallagher D, et al. Relationships between body roundness with body fat and visceral adipose tissue emerging from a new geometrical model. Obesity (Silver Spring, Md). 2013;21(11):2264–71. 23519954 10.1002/oby.20408 PMC3692604

[25] Wakabayashi I, Daimon T. The “cardiometabolic index” as a new marker determined by adiposity and blood lipids for discrimination of diabetes mellitus. Clin Chim Acta. 2015;438:274–8. 25199852 10.1016/j.cca.2014.08.042

[26] Amato MC, Giordano C, Galia M, Criscimanna A, Vitabile S, Midiri M, et al. Visceral Adiposity Index: a reliable indicator of visceral fat function associated with cardiometabolic risk. Diabetes Care. 2010;33(4):920–2. doi: 10.2337/dc09-1825 20067971 PMC2845052

[27] Kahn HS. The “lipid accumulation product” performs better than the body mass index for recognizing cardiovascular risk: a population-based comparison. BMC Cardiovasc Disord. 2005;5:26. 16150143 10.1186/1471-2261-5-26 PMC1236917

[28] Dobiásová M, Frohlich J. The new atherogenic plasma index reflects the triglyceride and HDL-cholesterol ratio, the lipoprotein particle size and the cholesterol esterification rate: changes during lipanor therapy. Vnitr Lek. 2000;46(3):152–6. 11048517

[29] Simental-Mendía LE, Rodríguez-Morán M, Guerrero-Romero F. The product of fasting glucose and triglycerides as surrogate for identifying insulin resistance in apparently healthy subjects. Metab Syndr Relat Disord. 2008;6(4):299–304. doi: 10.1089/met.2008.0034 19067533

[30] Williams KV, Erbey JR, Becker D, Arslanian S, Orchard TJ. Can clinical factors estimate insulin resistance in type 1 diabetes? Diabetes. 2000;49(4):626–32. doi: 10.2337/diabetes.49.4.626 10871201

[31] Zipf G, Chiappa M, Porter KS, Ostchega Y, Lewis BG, Dostal J. National health and nutrition examination survey: plan and operations, 1999-2010. Vital Health Stat 1. 2013;(56):1–37. 25078429

[32] Zheng X, Han W, Li Y, Jiang M, Ren X, Yang P, et al. Changes in the estimated glucose disposal rate and incident cardiovascular disease: two large prospective cohorts in Europe and Asia. Cardiovasc Diabetol. 2024;23(1):403. doi: 10.1186/s12933-024-02485-8 39511639 PMC11545867

[33] Peng J, Li A, Yin L, Yang Q, Pan J, Yi B. Estimated Glucose Disposal Rate Predicts Renal Progression in Type 2 Diabetes Mellitus: A Retrospective Cohort Study. J Endocr Soc. 2023;7(7):bvad069. doi: 10.1210/jendso/bvad069 37304203 PMC10251298

[34] Sun J, Wang N, Li S, Li M, Zhang A, Qin B, et al. Estimated glucose disposal rate and risk of arterial stiffness and long-term all-acuse mortality: a 10-year prospective study. J Epidemiol Comm Health. 2023;78(3). doi: 10.1136/jech-2023-220664 38123967

[35] Penno G, Solini A, Orsi E, Bonora E, Fondelli C, Trevisan R, et al. Insulin resistance, diabetic kidney disease, and all-cause mortality in individuals with type 2 diabetes: a prospective cohort study. BMC Med. 2021;19(1):66. doi: 10.1186/s12916-021-01936-3 33715620 PMC7962330

[36] Hajihashemi P, Mohammadifard N, Bateni M, Haghighatdoost F, Boshtam M, Najafian J, et al. Comparing the association of novel Anthropometric and atherogenicity indices with all-cause, cardiovascular and non-cardiovascular mortality in a general population of Iranian adults. Am J Prev Cardiol. 2025;21:100936. doi: 10.1016/j.ajpc.2025.100936 39967963 PMC11833613

[37] Nagayama D, Watanabe Y, Yamaguchi T, Suzuki K, Saiki A, Fujishiro K, et al. Issue of Waist Circumference for the Diagnosis of Metabolic Syndrome Regarding Arterial Stiffness: Possible Utility of a Body Shape Index in Middle-Aged Nonobese Japanese Urban Residents Receiving Health Screening. Obes Facts. 2022;15(2):160–9. doi: 10.1159/000520418 35008086 PMC9021625

[38] Zhang R, Hong J, Wu Y, Lin L, Chen S, Xiao Y. Joint association of triglyceride glucose index (TyG) and a body shape index (ABSI) with stroke incidence: a nationwide prospective cohort study. Cardiovasc Diabetol. 2025;24(1):7. 39762919 10.1186/s12933-024-02569-5 PMC11705842

[39] He H-M, Xie Y-Y, Chen Q, Li Y-K, Li X-X, Mu Y-K, et al. The additive effect of the triglyceride-glucose index and estimated glucose disposal rate on long-term mortality among individuals with and without diabetes: a population-based study. Cardiovasc Diabetol. 2024;23(1):307. doi: 10.1186/s12933-024-02396-8 39175051 PMC11342524

[40] Wilson PW, D’Agostino RB, Levy D, Belanger AM, Silbershatz H, Kannel WB. Prediction of coronary heart disease using risk factor categories. Circulation. 1998;97(18):1837–47. doi: 10.1161/01.cir.97.18.1837 9603539

[41] Yoshida M, Mita T, Yamamoto R, Shimizu T, Ikeda F, Ohmura C, et al. Combination of the Framingham risk score and carotid intima-media thickness improves the prediction of cardiovascular events in patients with type 2 diabetes. Diabetes Care. 2012;35(1):178–80. doi: 10.2337/dc11-1333 22028278 PMC3241317

[42] Park MJ, Choi KM. Association between Variability of Metabolic Risk Factors and Cardiometabolic Outcomes. Diabetes Metab J. 2022;46(1):49–62. doi: 10.4093/dmj.2021.0316 35135078 PMC8831817

[43] Poznyak A, Grechko AV, Poggio P, Myasoedova VA, Alfieri V, Orekhov AN. The Diabetes Mellitus-Atherosclerosis Connection: The Role of Lipid and Glucose Metabolism and Chronic Inflammation. Int J Mol Sci. 2020;21(5):1835. doi: 10.3390/ijms21051835 32155866 PMC7084712

[44] Yuan T, Yang T, Chen H, Fu D, Hu Y, Wang J, et al. New insights into oxidative stress and inflammation during diabetes mellitus-accelerated atherosclerosis. Redox Biol. 2019;20:247–60. doi: 10.1016/j.redox.2018.09.025 30384259 PMC6205410

[45] Agbaje AO, Barker AR, Mitchell GF, Tuomainen TP. Effect of arterial stiffness and carotid intima-media thickness progression on the risk of dysglycemia, insulin resistance, and dyslipidemia: a temporal causal longitudinal study. Hypertension. 2022;79(3):667–78. 35038890 10.1161/HYPERTENSIONAHA.121.18754 PMC8823909

[46] Silveira Rossi JL, Barbalho SM, Reverete de Araujo R, Bechara MD, Sloan KP, Sloan LA. Metabolic syndrome and cardiovascular diseases: Going beyond traditional risk factors. Diabetes Metab Res Rev. 2022;38(3):e3502. doi: 10.1002/dmrr.3502 34614543

[47] Velagapudi S, Karsai G, Karsai M, Mohammed SA, Montecucco F, Liberale L, et al. Inhibition of de novo ceramide synthesis by sirtuin-1 improves beta-cell function and glucose metabolism in type 2 diabetes. Cardiovasc Res. 2024;120(11):1265–78. doi: 10.1093/cvr/cvae100 38739545

[48] Yanai H, Adachi H, Hakoshima M, Iida S, Katsuyama H. Metabolic-Dysfunction-Associated Steatotic Liver Disease-Its Pathophysiology, Association with Atherosclerosis and Cardiovascular Disease, and Treatments. Int J Mol Sci. 2023;24(20). 10.3390/ijms242015473 PMC10607514 37895151

[49] Tanaka M. Improving obesity and blood pressure. Hypert Res. 2020;43(2):79–89. 10.1038/s41440-019-0348-x 31649313

[50] Hall JE, Mouton AJ, da Silva AA, Omoto ACM, Wang Z, Li X, et al. Obesity, kidney dysfunction, and inflammation: interactions in hypertension. Cardiovasc Res. 2021;117(8):1859–76. doi: 10.1093/cvr/cvaa336 33258945 PMC8262632

