# Supplementary figures and images for: The additive effect of the estimated glucose disposal rate and a body shape index on cardiovascular disease: A cross-sectional study
Source: PLoS One. 2025 Aug 21;20(8):e0331005. doi: 10.1371/journal.pone.0331005 (PMC12370132; doi:10.1371/journal.pone.0331005)

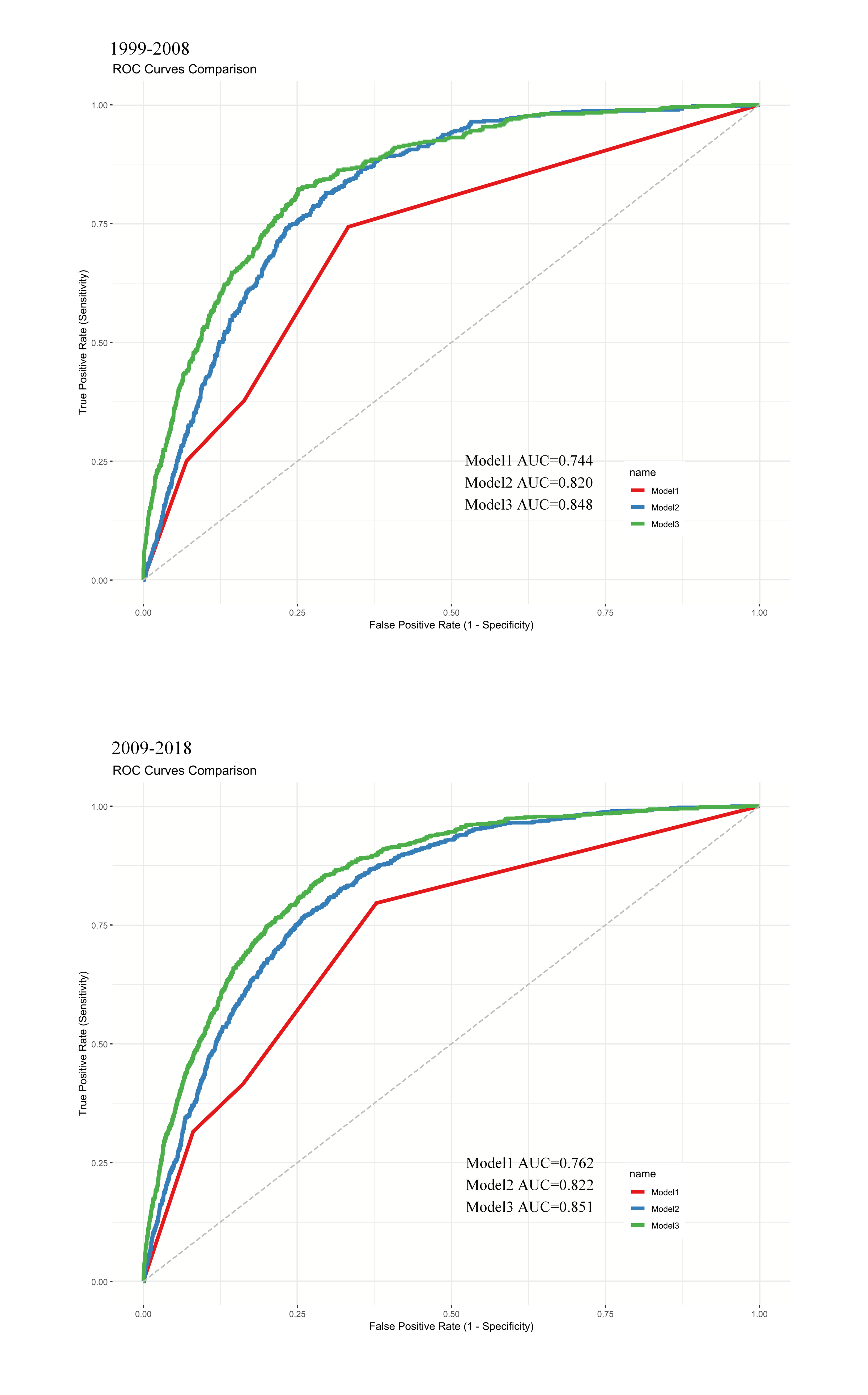

Supplement: S1 Fig — (TIF) [file pone.0331005.s002.tif]
